# A PAST YEAR OF CHALLENGE

**DOI:** 10.1016/S1808-8694(15)31037-5

**Published:** 2015-10-19

One year ago, we had the honor of writing our first Editorial for the Brazilian Journal of Otorhinolaryngology, entitled “The Challenge of the Past”. The text attempted to convey the responsibility of managing the Publications Department of our Journal, and continue this long and successful tradition of our major means of scientific disclosure. We thought we could anticipate the challenge we would have ahead of us. However, it was only after one year of work that we could appreciate the true size of such challenge and the repercussions of our actions.

During this first year, we tried to implement editorial and administrative changes in our journal. The first and foremost administrative measure was to centralize the stages of editing, formatting and printing management all in one single company, and this brought about an incredible speed to all the stages of our “production line”, with a substantial reduction (of about 45%) in lead time between paper submission and approval. Moreover, such measure is very much in tune with ABORL-CCF management goals and paramount for the financial balance of our Publications Department, and consequently, for that of our institution. In these regards, we also achieved an important increase in publicity space, of more than 30 %.

Late last year, our journal was indexed by the MedLine database, a final touch to the efforts of the previous administration, and this led to an important leap in the disclosure of our papers. Just to illustrate this fact, we have recorded about 25,000 hits per week in our Internet site in Portuguese, and about 1,700 hits in our site in English. And, finally, another action of great impact has been the updating of our journal at the National Library (Biblioteca Nacional), that requires, by Federal Law, since 2004, the submittal of all the issues of any publication printed in our country. In order to meet this challenge, we counted on the support of some colleagues, who have kindly donated some past issues that we no longer had in our library. We appreciate it and deeply thank them for this.

From the editorial stand point, the first change is right at the face. The new cover layout, more sober, tried to follow on the modern trend of important international journals, highlighting one image from the ones present in the papers that make the journal body. For that, we count on the outstanding scientific quality of the papers submitted and their images, which reflect the competence of Brazilian Otorhinolaryngologists in the production of scientific knowledge. The “Letter to the Editor” section has been an important asset introduced by our chief editor, allowing better interaction between subscribers, journal staff and authors – making room for productive discussions on controversial themes. The expansion of our editorial body, coupled to new case report submission standards, have also contributed to further enhance our editorial process.

And finally, the last novelty the authors enjoyed was to turn the Proceedings of our our Conventions into a scientific supplement to our journal. From now on, in selection processes, in competitive exams and, mainly, for research grant agencies and post-graduation programs, the summary of papers we present in our annual meetings will enjoy the status of being indexed in Exerpta Medica, Lilacs, SciELO and MedLine. And, there is much more to come.

And in closing, we would like to especially thank our Associate Publisher, Prof. Dr. Regina Helena G. Martins, with whom we have shared the pleasure of working in our department; our Chief Editor, Prof. Dr. João Ferreira de Mello Jr., who have strenuously dedicated himself to our journal; our associate editors, greatly responsible for the final scientific quality of each issue; our secretary Fernanda Vieira, who works hard in numerous activities; GN1 Genesis Network, most especially Márcio Argachof and Daniel Marcoto, who have truly “embodied our Journal” and, most especially, all the authors and co-authors who represent the main success pillars or our dear publication.

Silvio Caldas Neto

Publisher

